# Draft genome sequencing of the sugarcane hybrid SP80-3280

**DOI:** 10.12688/f1000research.11859.2

**Published:** 2017-07-03

**Authors:** Diego Mauricio Riaño-Pachón, Lucia Mattiello

**Affiliations:** 1 Brazilian Bioethanol Science and Technology Laboratory (CTBE), Brazilian Center for Research in Energy and Materials (CNPEM), Campinas, SP, Brazil; 2 Current address: Laboratory of Regulatory Systems Biology, Department of Biochemistry, Institute of Chemistry, University of São Paulo, São Paulo, SP, Brazil; 3 Current address: Functional Genome Laboratory, Department of Genetics, Evolution and Bioagents, Institute of Biology, State University of Campinas, Campinas, SP, Brazil

**Keywords:** sugarcane, long reads, polyploid, genomics

## Abstract

Sugarcane commercial cultivar SP80-3280 has been used as a model for genomic analyses in Brazil. Here we present a draft genome sequence employing Illumina TruSeq Synthetic Long reads. The dataset is available from NCBI BioProject with accession PRJNA272769.

## Introduction

Sugarcane is an economically important crop used as source of sugar, ethanol and electricity generation ^1^. Sugarcane has a haploid genome of ~1Gpb, however, modern sugarcane cultivars are polyploids derived from interspecific hybridization between *S. officinarum* L. and *S. spontaneum* L., reaching up to 130 chromosomes distributed among ~12 homo(eo)logous groups ^2, 3^, with a total genome size reaching 10Gpb ^4^. Its complex genome structure has hampered genome sequencing, assembly and annotation. Partial genomic sequences are available ^5– 8^, as well as transcriptome sequences ^9– 11^, but there are no whole genome assemblies available to date. Here we used the Illumina TruSeq Synthetic Long Read sequencing technology to survey the genome of the polyploid cultivar SP80-3280. The generated long reads, their assembly and genome annotation have been made public and will provide useful information for functional genomics studies.

## Materials and methods

The leaf rolls of greenhouse grown, two-month old plants of sugarcane cultivar SP80-3280 (provided by Centro de Tecnologia Canavieira, Piracicaba, São Paulo), were collected and immediately frozen in liquid nitrogen. The plant tissue was ground up to become fine powder, and high molecular weight DNA was extracted from 100 mg of fresh frozen tissue using CTAB (Sigma-Aldrich, USA) and chloroform:isoamyl alcohol (Sigma-Aldrich, USA) as previously described ^12^. 6µg of DNA were sent to Illumina (CA, USA) for DNA sequencing using TruSeq Synthetic long read technology ^13^, through their FastTrack Sequencing Service. Sequencing was performed on an Illumina HiSeq2000 system using paired-end chemistry. Nine long read libraries, each generating approx. 600Mbps, were generated, giving an estimated coverage between 4 and 5 of the monoploid genome. A total of 1,378,917 reads longer than 1.5Kbp, or 5,642,855,018 bases, were generated. The underlying 1,966,604,928 short reads amount to 393,320,985,600bp, which would translate to an estimated coverage of 393x of the haploid genome. The maximum read length was 20,918bp, with 36% of the reads being longer than 4.5Kbp. Possible contaminants were removed by comparison against the NCBI’s nucleotide database using BLAST ^14^, keeping only the long reads with best hits against Viridiplantae, resulting in 1,224,061 useful for assembly. Prior to assembly, long reads originating from mitochondria (NC_008360.1) and chloroplast (NC_005878.2) were excluded using mirabait ( http://mira-assembler.sourceforge.net/). Reads longer than 1.5Kbp were assembled using Celera’s WGS Assembler v8.2 ^15^, using similar parameters as previously described ^13^, except for some of the error parameters that were left in their default settings, i.e., ‘unitiger=bogart, merSize=31, ovlMinLen=100’, and the parameters ovlErrorRate, cnsErrorRate, cgwErrorRate, utgGraphErrorRate, utgGraphErrorLimit, utgMergeErrorRate, utgMergeErrorLimit. A non-redundant assembly was created using CD-HIT ^16^, merging 100% identical sequences and sub-sequences. RNASeq data previously generated in our group ^17^ for the same cultivar was exploited for gene prediction using BRAKER1 ^18^ and PASA ^19^, as well as sugarcane transcript data (ESTs), and *Sorghum bicolor* proteins using Exonerate ^20^, all gene evidence was integrated to generate a high quality gene prediction set with Evidence Modeller ^21^, leading to 153,078 predicted protein-coding genes.

## Data availability

The data referenced by this article are under copyright with the following copyright statement: Copyright: © 2017 Riaño-Pachón DM and Mattiello L

Raw sequencing data are available at NCBI SRA; the long reads with accession number SRX845504, and the underlying short reads with accessions SRX853961 to SRX853969. The SP80-3280 assembly is available with accession number GCA_002018215.1. All data can be found under the BioProject PRJNA272769. Genome annotation is available from https://figshare.com/projects/Sugarcane_SP80-3280_draft_genome_annotation/22327
