# Supplementary material for: Draft genome sequencing of the sugarcane hybrid SP80-3280
Source: F1000Res. 2017 Jul 3;6:861. Originally published 2017 Jun 9. [Version 2] doi: 10.12688/f1000research.11859.2 (PMC5499785; doi:10.12688/f1000research.11859.2)
Supplement: Peer review history

# Peer review: Referee response for version 1

**DOI:** 10.5256/f1000research.12814.r23667

**Published:** 2017-06-21

**Authors:** Chakravarthi Mohan

**Affiliations:** 1 Department of Genetics and Evolution, Federal University of São Carlos, São Carlos, Brazil

The data note entitled ' Draft genome sequencing of the sugarcane hybrid SP80-3280' is perhaps the first report describing the whole genome of sugarcane, a complex polyploid and its availability in NCBI will be a boon to sugarcane researchers.

 The study is well planned, executed and well drafted. The data presented here would be particularly useful for functional genomic studies in sugarcane.

I have read this submission. I believe that I have an appropriate level of expertise to confirm that it is of an acceptable scientific standard.

# Response

**Published:** 2017-06-23

**Authors:** Diego Mauricio Riaño-Pachón

**Affiliations:** Molecular Biology, University of São Paulo, Brazil

Dear Dr. Mohan,

 thanks you for your review of our data note. In version 2 of the note we have added links for the genome annotation in addition to the genome assembly.

 Best regards,

 Diego

# Peer review: Referee response for version 1

**DOI:** 10.5256/f1000research.12814.r23398

**Published:** 2017-06-15

**Authors:** Jason Miller

**Affiliations:** 1 J. Craig Venter Institute, Rockville, MD, USA

Summary:

 The Data Note, "Draft genome sequencing of the sugarcane hybrid SP80-3280", describes a sugarcane genome assembly that is available at NCBI. The TruSeq method was applied to a monoploid sugarcane cultivar to generate a 1.2 gigabase assembly with a 8433 contig N50 according to GenBank. This is the first sugarcane genome assembly so it will be of interest to the field. This data note is especially useful because it describes the sequence filtering by size, blast, mirabit, and cd-hit prior to release.

 Suggestions:

 The sentence, “there are not whole genome assemblies available”, probably should say “there are no whole genome assemblies available”. The text could be made clearer by presenting all the statics for underlying short reads before getting to the synthetic long read stats, and by specifying that the blast filter was applied to the long reads. I would appreciate a reference for Celera Assembler, but that is just me.

I have read this submission. I believe that I have an appropriate level of expertise to confirm that it is of an acceptable scientific standard.

# Response

**Published:** 2017-06-23

**Authors:** Diego Mauricio Riaño-Pachón

**Affiliations:** Molecular Biology, University of São Paulo, Brazil

Dear Dr. Miller,

 thank you very much for your review of our data note. We have followed your main suggestions, and they are available as version 2 of the data note.

 Best regards,

 Diego
